# A gene expression signature in HER2+ breast cancer patients related to neoadjuvant chemotherapy resistance, overall survival, and disease-free survival

**DOI:** 10.3389/fgene.2022.991706

**Published:** 2022-10-21

**Authors:** Carlos A. Barrón-Gallardo, Mariel Garcia-Chagollán, Andres J. Morán-Mendoza, Raul Delgadillo-Cristerna, María G. Martínez-Silva, María M. Villaseñor-García, Adriana Aguilar-Lemarroy, Luis F. Jave-Suárez

**Affiliations:** ^1^ Programa de Doctorado en Ciencias Biomédicas, Centro Universitario de Ciencias de La Salud, Universidad de Guadalajara, Guadalajara, Mexico; ^2^ Instituto de Investigación en Ciencias Biomédicas (IICB), Centro Universitario de Ciencias de La Salud, Universidad de Guadalajara, Guadalajara, Mexico; ^3^ Hospital de Ginecología, Centro Médico Nacional de Occidente, Instituto Mexicano Del Seguro Social (IMSS), Guadalajara, Mexico; ^4^ Departamento de Radiología e Imagen, Centro Médico Nacional de Occidente, Instituto Mexicano Del Seguro Social (IMSS), Guadalajara, Mexico; ^5^ Departamento de Anatomía Patológica, Centro Médico Nacional de Occidente, Instituto Mexicano Del Seguro Social (IMSS), Guadalajara, Mexico; ^6^ División de Inmunología, Centro de Investigación Biomédica de Occidente, Instituto Mexicano Del Seguro Social (IMSS), Guadalajara, Mexico

**Keywords:** breast cancer, neoadjuvant chemotherapy, RNA-seq, biomarkers, bioinformatics, overall survival, disease free survival

## Abstract

Breast cancer ranks first in terms of mortality and incidence rates worldwide among women. The HER2+ molecular subtype is one of the most aggressive subtypes; its treatment includes neoadjuvant chemotherapy and the use of a HER2 antibody. Some patients develop resistance despite positive results obtained using this therapeutic strategy. Objective. To identify prognostic markers for treatment and survival in HER2+ patients. Methods. Patients treated with neoadjuvant chemotherapy were assigned to sensitive and resistant groups based on their treatment response. Differentially expressed genes (DEGs) were identified using RNA-seq analysis. KEGG pathway, gene ontology, and interactome analyses were performed for all DEGs. An enrichment analysis Gene set enrichment analysis was performed. All DEGs were analyzed for overall (OS) and disease-free survival (DFS). Results. A total of 94 DEGs were related to treatment resistance. Survival analysis showed that 12 genes (ATF6B, DHRS13, DIRAS1, ERAL1, GRIN2B, L1CAM, IRX3, PRTFDC1, PBX2, S100B, SLC9A3R2, and TNXB) were good predictors of disease-free survival, and eight genes (GNG4, IL22RA2, MICA, S100B, SERPINF2, HLA-A, DIRAS1, and TNXB) were good predictors of overall survival (OS). Conclusion: We highlighted a molecular expression signature that can differentiate the treatment response, overall survival, and DFS of patients with HER2+ breast cancer.

## Introduction

Breast cancer is a heterogeneous disease characterized by abnormal and uncontrolled growth of malignant breast cells. Among all types of cancer, this disease ranks first in mortality and incidence rates in women over 25 years of age worldwide (Sung et al., 2021). In 2000, Perou et al. reported different molecular expression patterns in patients with breast cancer, and these patterns were subsequently used to classify breast cancer into distinct molecular subtypes (Perou et al., 2000; Sorlie et al., 2003). According to this classification, cancer cells that express human epidermal growth factor 2 (ERBB, formerly HER2) and not estrogen receptors (ER) are identified as the HER2+ molecular subtype, which represents 15%–30% of breast cancer patients, is an aggressive phenotype, and a predictor of poor outcome (Ban et al., 2020).

The treatment of HER2+ breast cancer includes the administration of chemotherapy and trastuzumab, a monoclonal antibody against the HER2 receptor (Abal et al., 2003; Harbeck and Gnant, 2017; Waks and Winer, 2019). Conventional neoadjuvant chemotherapy involves anthracyclines followed by taxane application. Anthracyclines work by joining DNA and suppressing the binding of DNA polymerase, thereby preventing DNA replication (McGowan et al., 2017). Taxanes affect mitotic spindle formation by binding to tubulin dimers, thereby preventing the division of tumor cells (Yardley, 2013; Harbeck and Gnant, 2017). Furthermore, adding trastuzumab in conventional chemotherapy helps block HER2 receptor-induced cell growth signaling (Maximiano et al., 2016). Despite the positive results obtained with this therapeutic strategy, some patients develop resistance. The molecular mechanisms underlying resistance are not fully understood; therefore, there is a lack of predictive biomarkers that are helpful in the prognosis and prediction of chemotherapy response (Iwamoto et al., 2020).

This study aimed to evaluate the transcriptome of HER2+ breast cancer patients and, according to their response to chemotherapy (sensitivity or resistance), to identify differentially expressed genes (DEGs) that could be useful in predicting patient outcomes after neoadjuvant chemotherapy treatment.

## Materials and methods

### Sample selection, chemotherapy treatment, and study design

Patients aged 18 years and older with a diagnosis of breast cancer, HER2+/PR-/ER-, tumor size >2 cm, and positive nodes, candidates to receive neoadjuvant chemotherapy, and without previous therapy against cancer were recruited for this study. Patients with metastatic cancer, those with insufficient breast cancer biopsy tissue for pathological analysis, or those with RNA extraction were excluded. All participants provided written informed consent prior to enrolment. The data were deposited in the Gene Expression Omnibus (GEO) repository under the number GSE162187. Samples were separated by pathologic response into two groups: pathological complete response (pCR) was considered the sensitive group, and those in the non-pCR group were considered the resistant group.

Additionally, we used and analyzed data from the GSE163882 study, and HER2+/PR-/ER-samples were selected. The results obtained from both databases were compared.

Finally, from the TCGA breast ductal carcinoma database, HER+/PR-/ER-breast cancer samples were selected for analysis of overall survival.

### Ethics and informed consent statements

The study was conducted in accordance with the guidelines of the Declaration of Helsinki and the ethical standards of the institutional and/or national research committee. This study was approved by the Ethical and Research Committee of the Instituto Mexicano del Seguro Social (IMSS) (number R-2013-785-061). Informed consent was obtained from all subjects involved in the study.

### Quality control, alignment, and differential expression

The FASTQ files were analyzed with the Flexbar software tool version 3.5.0 (https://github.com/seqan/flexbar/releases/tag/v3.5.0) (Dodt et al., 2012; Roehr et al., 2017) to remove Illumina adapters and to filter reads by a Phred score >30. To quantify the RNA-seq data, a pseudo-alignment was performed using Kallisto software version 0.46.1 (https://pachterlab.github.io/kallisto/download.html) (Bray et al., 2016) with the default parameters and the GRCh38 human genome reference (GRCh38. p12). The DESeq2 package version 1.28.1 (https://bioconductor.riken.jp/packages/3.0/bioc/html/DESeq2.html) (Love et al., 2014) was used for the analysis of abundance tables and the identification of differentially expressed genes (DEGs) for comparing resistant and sensitive samples (set as the reference group). The Ensembl database was used for the annotation of genes. To decrease the false discovery rate, the Benjamini–Hochberg correction test was applied to obtain adjusted *p*-values.

### Enrichment and interaction analysis

We selected all DEGs (*p* < 0.05) obtained from GSE162187 for analysis with the KEGG Mapper (https://www.genome.jp/kegg/tool/map_pathway1.html) (Kanehisa and Sato, 2020) and the DAVID v6.8 web tools (https://david.ncifcrf.gov/home.jsp) to identify pathways implicated in treatment response.

The Panther database v.16.0 (Mi et al., 2021) web tool was used for Gene Ontology enrichment analysis using Fisher’s exact test and false discovery rate (FDR), with a threshold of *p* < 0.05, which was considered to be significant for each of the three categories, that is, molecular function, cellular component, and biological process.

Gene set enrichment analysis (GSEA) was performed using the pre-ranked DEGs list. GSEA software v4.2.2 was used for analysis (Subramanian et al., 2005). A molecular signature database (MsigDB v7.4) was used, taking the nine collections (C1:C9, and H) for enrichment analysis (Subramanian et al., 2005; Liberzon et al., 2011; Liberzon et al., 2015).

To perform an interactome analysis, the DEGs were filtered by adjusted *p*-value <0.05 and analyzed using STRING-DB v11.0 software (https://string-db.org/) (Szklarczyk et al., 2019), the confidence score was set up at 0.7 to represent protein–protein associations.

### Principal component analysis and heatmap representation

The geometric mean of the counts for each gene was used as a normalization factor. Once normalized, the principal component analysis (PCA) and heatmap representation were performed with the prcomp package and heatmap functions, respectively, with the default parameters in R v.4.0.2 (“Taking off Again”) using as variables the normalized counts of the DEGs with an adjusted *p*-value of <0.05.

### Survival analysis

Furthermore, a database of 109 patients obtained from the TCGA breast ductal carcinoma study with HER+/ER-/PR-was analyzed (TCGA Research Network: https://www.cancer.gov/tcga) at 60 and 120 months to analyze overall survival (OS) and disease-free survival (DFS). Gene expression levels were determined according to normalized Log2-read counts for each gene. Median and quartiles were used for determining high- and low-expression groups. DEGs with an adjusted *p*-value < 0.05 were analyzed. Statistical significance was set at *p* < 0.05.

## Results

A previous study was conducted to determine biomarkers of response to neoadjuvant chemotherapy in patients with breast cancer (GSE162187) (Barron-Gallardo et al., 2022). In this study, HER2+ samples were taken and included in the RNA-seq analysis; five samples were from patients categorized as resistant to treatment, and three samples were from patients sensitive to treatment. This small subset was used as training data. The results were validated using the GSE163882 dataset, which included information from 222 patients with breast cancer. Patients were over 33 years old; the mean ages for the resistant and sensitive groups were 52.2 (±12.15) and 62 (±6.24) years, respectively, with no statistical differences. The diagnostic status of all the patients was invasive ductal carcinoma breast cancer. The histological grades for tumor biopsies based on SBR (Scarff–Bloom–Richardson) parameters were five SBRII, two SBRIII, and one with non-available information (Supplementary Table S1).

The transcriptomic pattern was studied to determine the variables that specifically discriminated HER2+ patients according to their neoadjuvant treatment response. Despite having two groups defined by their pathological response, principal component analysis (PCA) with all genes detected by RNA-seq showed that the samples did not form specific clusters. Moreover, the distribution of the samples followed a heterogeneous pattern, indicating that HER2+ breast cancer patients may have high variability in gene expression (Supplementary Figure S1A).

### Determination of DEGs related to treatment resistance in the training data

**FIGURE 1:**
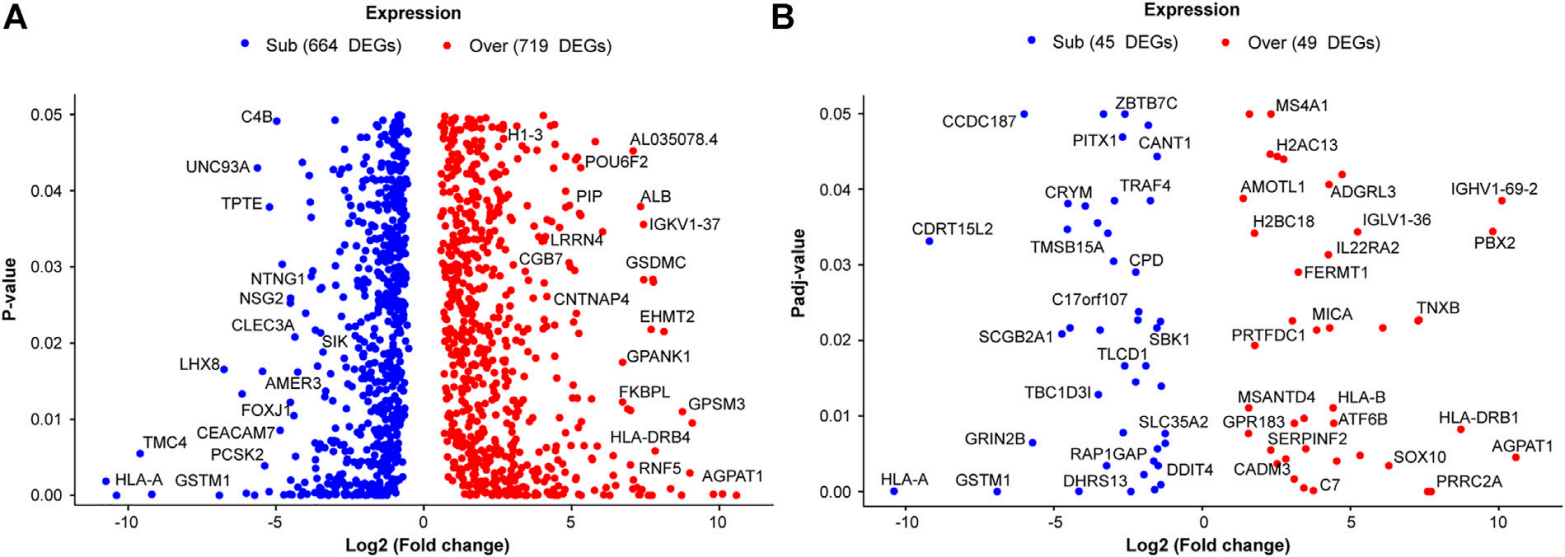
Differentially expressed genes (DEGs) related to neoadjuvant chemotherapy outcomes in HER2+ patients with breast cancer. Each point represents every DEG in the resistant group compared with the sensitive. Overexpressed genes are colored red, subexpressed genes are represented by blue points. The *x*-axis shows the value of the log2 (fold change), and the *y*-axis represents **(A)** the *p*-value and **(B)** the adjusted *p*-value.

HER2+ patients were grouped into sensitive and resistant to neoadjuvant chemotherapy groups to obtain DEGs related to treatment response. The transcriptomes of both groups were compared, and a total of 1383 genes were observed to be differentially expressed (*p* < 0.05), of which 719 were sub-expressed and 664 were overexpressed in the resistant group as compared with the sensitive group (Figure 1A). To diminish the inclusion of false-positive DEGs, the Benjamini–Hochberg post hoc test was applied; among the 1383 DEGs, only 94 maintained statistical significance with an adjusted *p*-value (p-adj) <0.05 (45 subexpressed and 49 overexpressed genes) (Figure 1B).

Thereafter, we investigated whether this set of 94 DEGs could adequately classify the samples as sensitive and resistant to treatment. Therefore, principal component analysis (PCA) with only 94 genes was performed again. The results showed two clusters defined by principal component 1 (PC1) with 55.35% and principal component 2 (PC2) with 15.79% of the data variance (Supplementary Figure S1B). Therefore, the selection of the 94 DEGs included genes capable of clustering HER2+ patients into resistant and sensitive groups. As shown in Supplementary Table S2, from the 94 DEGs, the top 10 overexpressed genes were HLA-DQA1, TRIM26, IGHJ6, AGPAT1, IGHV1-69-2, PBX2, HLA-DRB1, PRRC2A, LRRC37A3, and TNXB, and the top 10 underexpressed genes were HLA-A, CDRT15L2, GSTM1, CCDC187, GRIN2B, SCGB2A1, GNG4, SBSN, CRISP3, and ZG16B.

**FIGURE 2:**
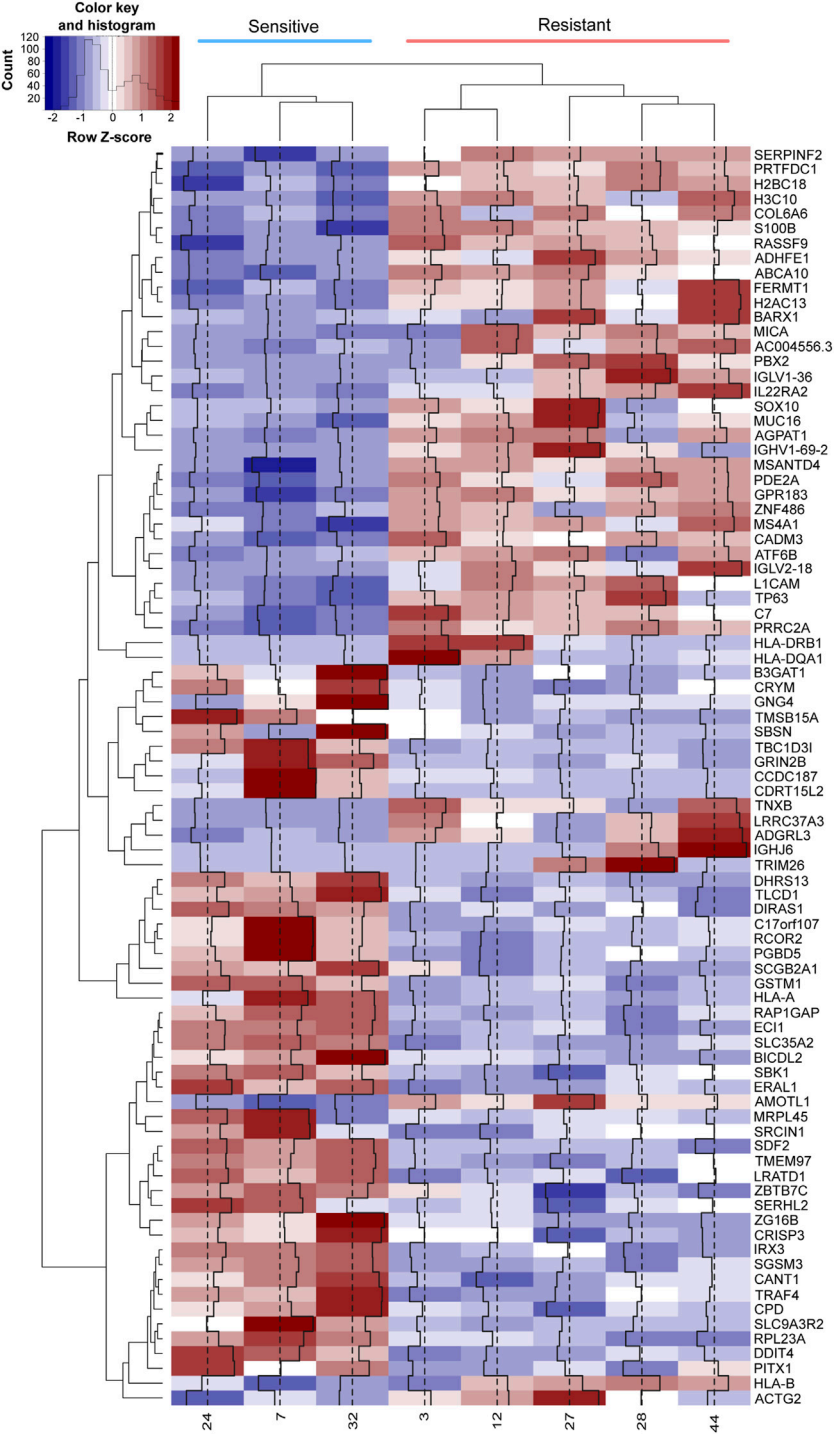
Expression patterns of DEGs in resistance and sensitivity. The row Z-score of the normalized read counts of DEGs with p-adj < 0.05 are plotted in the heatmap. The red color indicates a row Z-score >0, and the blue indicates a row Z-score <0. Columns represent each patient, and each row represents a gene. The dendrogram at the top of the heatmap clusters the patients according to their gene expression pattern, while the dendrogram at the left side of the heatmap groups the genes with similar expression patterns. Columns 1 to 3 represent sensitive patients, and columns 4 to 8 represent resistant patients.

To visualize the expression pattern (color density) and distribution (clustering) of the 94 DEGs, heatmap analysis was performed (Figure 2). The column dendrogram results showed two clusters belonging to the sensitive and resistant groups. The row dendrogram shows four clusters of genes with similar expression patterns.

### Pathways and enrichment analysis of DEGs related to chemotherapy resistance

**FIGURE 3:**
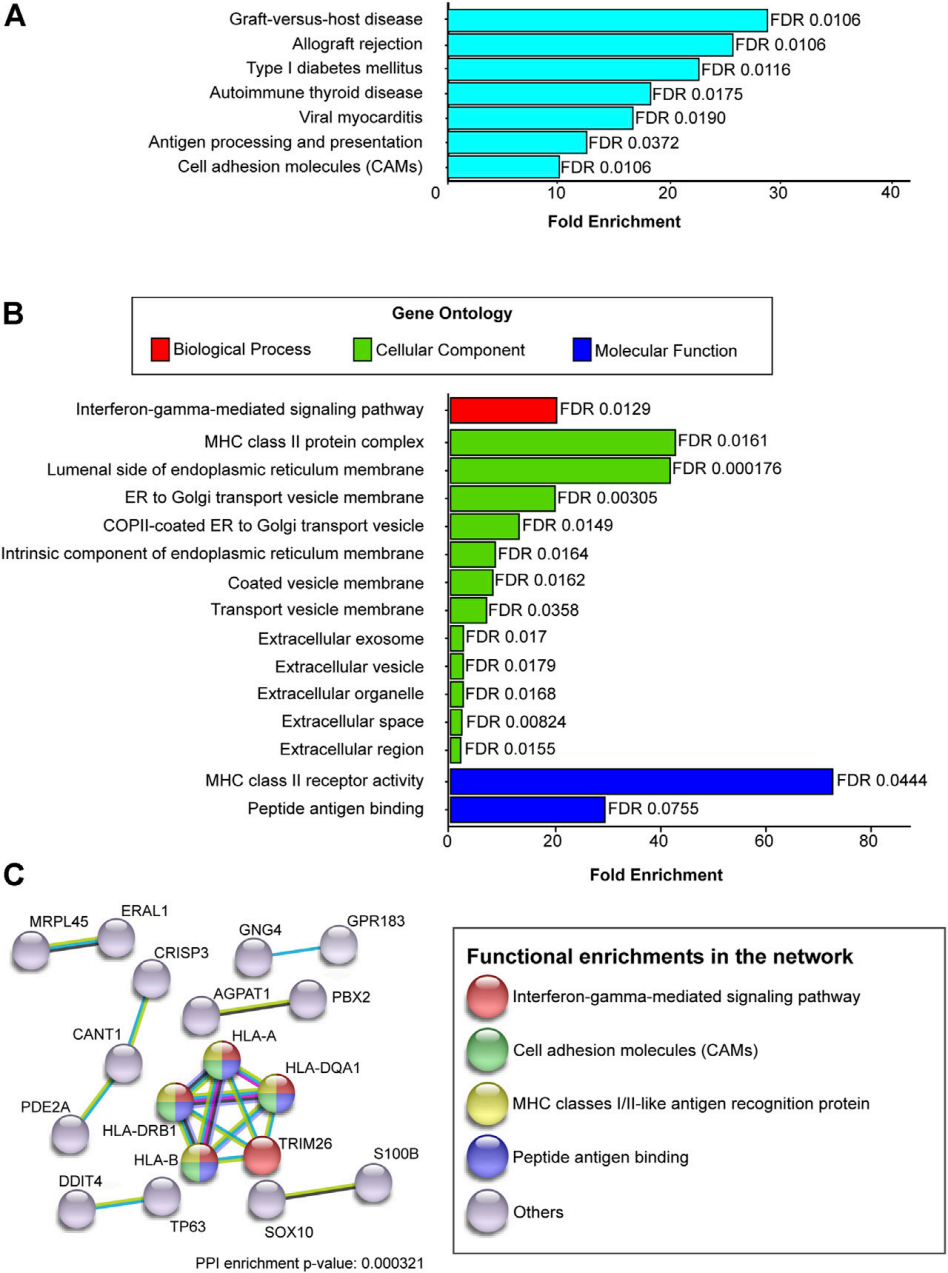
KEGG, GO, DEGs interaction related to chemotherapy resistance. The 94 DEGs with a p-adj < 0.05 were analyzed to know their contribution to KEGG pathways, gene ontology, and the interaction clusters **(A)** KEGG enrichment analysis **(B)** GO enrichment analysis. Each bar represents the fold enrichment value for KEGG and GO. The *x*-axis plots the fold enrichment values, and the *y*-axis shows the pathway’s name or GO terms. In GO enrichment analysis, the plot is divided into three categories: biological process (red bars), cellular component (green bars), and molecular function (blue bars). **(C)** Interactome analysis. Only DEGs that interact with each other were plotted in the graph. Network nodes represent proteins encoded by DEGs; colors represent the category to which encoded proteins belong; edges represent protein-protein interactions. Line colors indicate the type of interaction reported.

The 94 DEGs were analyzed using the KEGG Mapper search pathway tool and DAVID v6.8. Among the 318 KEGG pathways, seven were statistically modulated (FDR <0.05) (Figure 3A), including graft-versus-host disease, allograft rejection, type I diabetes mellitus, autoimmune thyroid disease, viral myocarditis, antigen processing and presentation, and cell adhesion molecules. In addition, GO analysis results showed that the biological processes enriched by DEGs were related to the interferon-gamma-mediated signaling pathway. The cellular components in which the DEGs were included were associated with the MHC class II protein complex, luminal side of the endoplasmic reticulum membrane, endoplasmic reticulum (ER)-to-Golgi transport vesicle membrane, and extracellular space. Finally, the modulated molecular functions were MHC class II receptor activity and peptide-antigen binding (Figure 3B).

Enrichment analysis showed a total of 40 gene sets enriched with a *p*-value < 0.05 (35 positively and five negatively), which belongs to C1 (1 enriched set), C2 (6 enriched sets), C3 (2 enriched sets), C5 (19 enriched sets), C7 (7 enriched sets), and C8 collections (5 enriched sets). The C4, C6, and H collections did not contain enriched sets. From the enriched sets, we found two related to therapy resistance (Massarweh tamoxifen resistance and Creighton endocrine therapy resistance gene sets) and three related to the immune system, such as GOBP immune response, Goldrath antigen response, and CHR6P21, which is a location for genes related to the immune system (HLA-DQA1, HLA-DRB1, HLA-B, and MICA) (Supplementary Table S2).

### Determination of interactions clusters between DEGs

An analysis of 94 DEGs was performed to determine the molecular interactions between them. The STRING-DB tool was used to set an interaction score with high confidence (0.7). The results showed 18 edges (genes) distributed among seven clusters: one with five genes, one with three genes, and five with two genes. Among the seven clusters, the cluster with five edges was related to the interferon-gamma-mediated signaling pathway, MHC class I/II-like antigen recognition protein, and cell adhesion molecules, including the HLA-A, HLA-DQA1, TRIM26, HLA-B, and HLA-DRB1 genes (Figure 3C).

### Evaluation of DEGs for survival prediction

**FIGURE 4:**
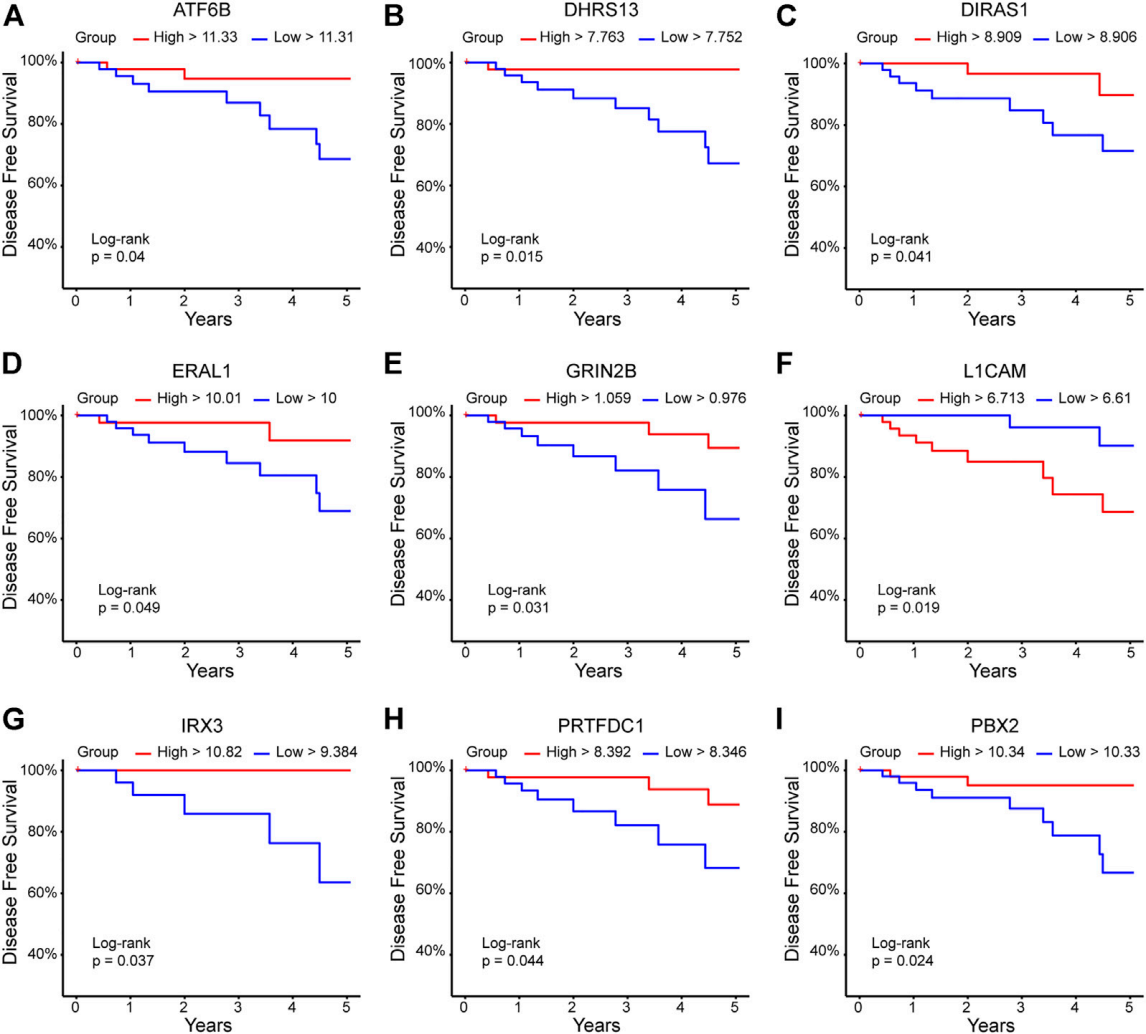
DEGs related to the prediction of DFS at 5 years. The 94 DEGs (p-adj<0.05) were analyzed in the TCGA ductal breast cancer database (https://www.cancer.gov/tcga). The red line indicates the high expression group, and the blue line represents the low expression group. *Y*-axis shows the DFS percentage; *X*-axis shows the time in years. **(A)** ATF6B, **(B)** DHRS13, **(C)** DIRAS1, **(D)** ERAL1, **(E)** GRIN2B, **(F)** L1CAM, **(G)** IRX3, **(H)** PRTFDC1, **(I)** PBX2.

**FIGURE 5:**
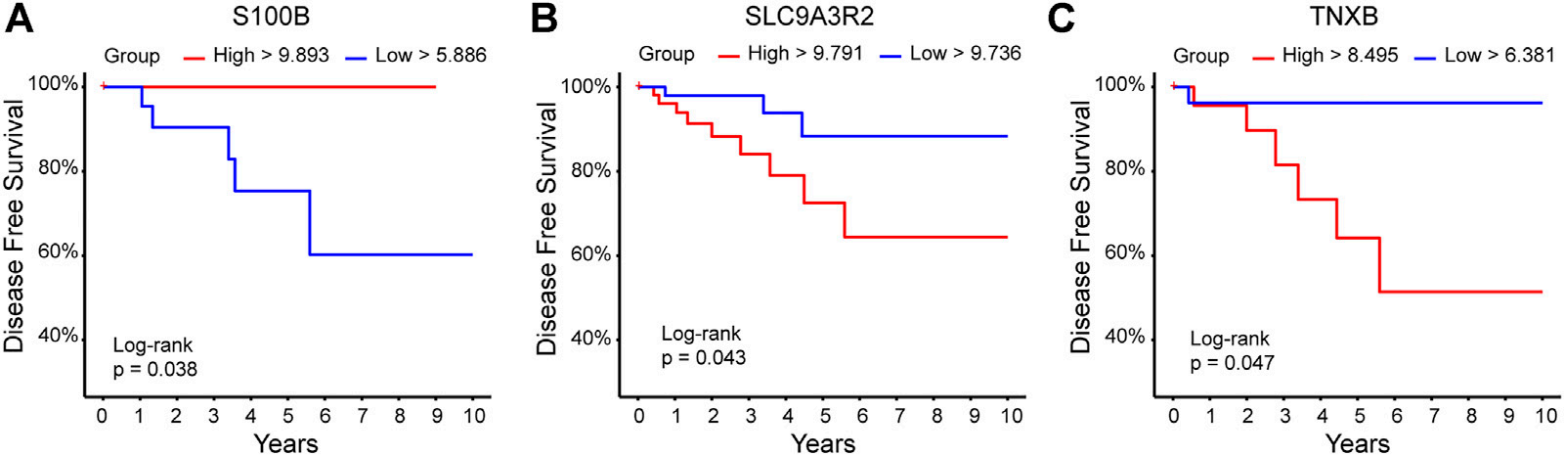
DEGs related to the prediction of DFS at 10 years. DEGs that meet the criteria of p-adj<0.05 were analyzed using the TCGA ductal breast cancer database (https://www.cancer.gov/tcga) to determine their association with DFS at 10 years. The red line indicates the high expression group, the blue line represents the low expression group. *Y*-axis shows the disease-free survival percentage; *X*-axis shows the time in years. **(A)** S100B, **(B)** SLC9A3R2, **(C)** TNXB.

To evaluate whether the expression of the 94 DEGs was related to survival prediction, measured as DFS or OS, we analyzed the 94 DEGs individually using a database with expression information of 109 patients with HER+/ER-/PR-, and data were obtained from TCGA breast ductal carcinoma study. A total of 12 DEGs predicted the DFS. The high expression of ATF6B, DHRS13, DIRAS1, ERAL1, GRING2B, IRX3, PRTFDC1, and PBX2 was found to be an excellent prognostic of DFS at 5 years; on the other hand, a high expression of L1CAM was associated with lower DFS at 5 years (Figure 4). We found that low expression of TNXB and SLC9A3R2 and high expression of S100B were associated with better DFS in the long term (10 years) (Figure 5).

**FIGURE 6:**
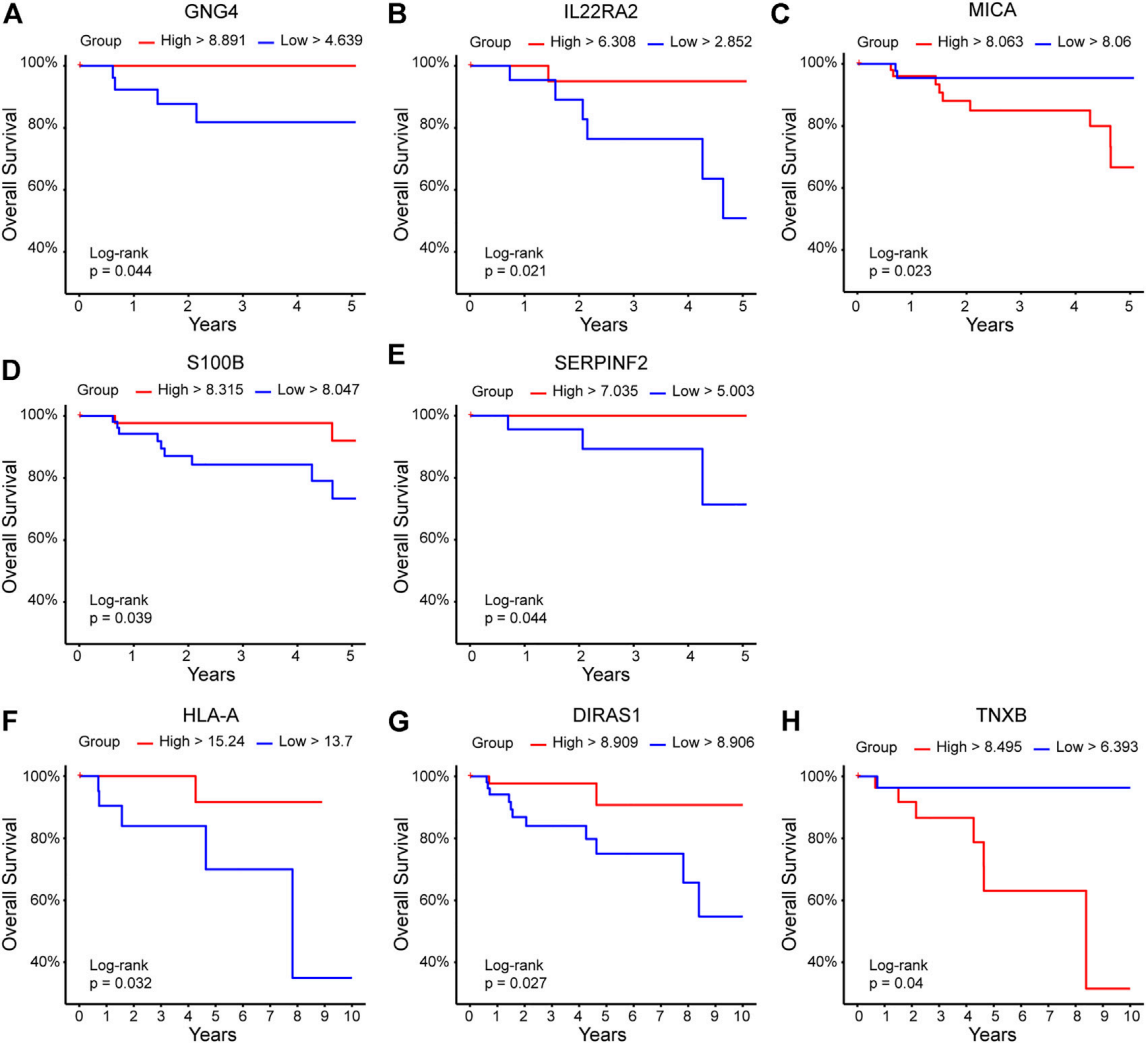
DEGs related to overall survival. DEGs with p-adj<0.05 were contrasted with overall survival data at 5 and 10 years in the TCGA ductal breast cancer database (https://www.cancer.gov/tcga). The red line indicates the high expression group, and the blue line represents the low expression group. *Y*-axis shows the overall survival percentage; *X*-axis shows the time in years. Panels for **(A)** GNG4, **(B)** IL22RA2, **(C)** MICA, **(D)** S100B, and **(E)** SERPINF2 represent OS at 5 years, while panels for **(F)** HLA-A, **(G)** DIRAS1, and **(H)** TNXB showed OS data for 10 years.

According to the OS analysis, groups with high expression of GNG4, IL22RA2, S100B, and SERPINF2 were associated with better OS at 5 years; the same was true for HLA-A and DIRAS1 at 10 years. In contrast, high expression of MICA and TNXB was related to lower OS times at 5 and 10 years, respectively (Figure 6).

Odds ratio analysis was performed to determine if there is any difference at the end point of five of 10 years in OS or DFS. The results of the odds ratio analysis showed a similar prognostic pattern for each gene compared with the results obtained from the log-rank analysis, except for GRIN2B, PRTFDC1, SLC9A3R2 in DFS and HLA-A, IL22RA2 in OS whose *p* values were greater than 0.05 (Supplementary Table S3).

Furthermore, univariate and multivariate Cox analyses were performed. The results show some genes in which the expression can be considered a predictor variable associated with survival time. In univariate cox analysis for DFS, the coefficients were negative for DHRS13, GRIN2B and positive for L1CAM with *p* < 0.05. When applying the univariate cox analysis for OS, DIRAS had a negative coefficient, and MICA had a positive coefficient *p* < 0.05 (Table 1). We performed multivariate cox analysis using age, pathologic stage, radiation therapy, and the expression level as variables. The results show that as higher the pathologic stage, the hazard to disease recurrence increases for ATF6B, DHRS13, DIRAS1, ERAL1, GRIN2B, L1CAM, PBX2, PRTFDC1, SLC9A3R2, and TNXB, furthermore, increase the risk of death when analyzed DIRAS1, IL22RA, MICA, and S100B. Alongside, neoadjuvant radiation therapy was correlated with decrease recurrence risk in ATF6B, DHRS13, DIRAS1, LICAM, and SLC9A3R2, and decreases dead risk when analyzed S100B, MICA, and DIRAS1 (Table 2).

**TABLE 1:** Univariate cox regression analysis for expression variable.

Gene	Variable	Coef	exp (coef)	se (coef)	z	Pr (>|z|)	Survival
DHRS13	Expression	-2.1257	0.1193	1.0494	-2.026	0.0428	DFS
GRIN2B	Expression	-1.3827	0.2509	0.6886	-2.008	0.0446	DFS
L1CAM	Expression	1.6424	5.1677	0.7829	2.098	0.0359	DFS
DIRAS1	Expression	-1.5443	0.2135	0.7695	-2.007	0.0448	OS
MICA	Expression	1.6142	5.0238	0.7847	2.057	0.0397	OS

Coef = coefficient; exp = Exponential; se = standard error.

**TABLE 2:** Multivariate Cox Regression Analysis including clinical variables

Survival type	Gene	Variable	Coef	exp (coef)	se (coef)	p-value
DFS	ATF6B	Pathologic_stage	0.56021	1.75105	0.19235	0.0036
		Radiation_therapy	-1.52251	0.21816	0.76688	0.0471
	DHRS13	Pathologic_stage	0.76044	2.13923	0.22323	0.0007
		Radiation_therapy	-1.56215	0.20968	0.71944	0.0299
	DIRAS1	Pathologic_stage	0.73385	2.08308	0.21171	0.0005
		Radiation_therapy	-1.64476	0.19306	0.72105	0.0225
	ERAL1	Pathologic_stage	0.67782	1.96958	0.19343	0.0005
	GRIN2B	Pathologic_stage	0.71306	2.04023	0.21839	0.0011
	L1CAM	Pathologic_stage	0.56388	1.75748	0.17428	0.0012
		Radiation_therapy	-1.95488	0.14158	0.74922	0.0091
	PBX2	Pathologic_stage	0.57663	1.78003	0.19418	0.003
	PRTFDC1	Expression	-2.26336	0.104	1.10961	0.0414
		Pathologic_stage	0.64406	1.90419	0.1972	0.0011
	S100B	Expr_quant	3.647	38.37	1.088	0.0008
	SLC9A3R2	Expression	3.08808	21.9349	1.21697	0.0112
		Pathologic_stage	0.51109	1.66711	0.18757	0.0064
		Radiation_therapy	-2.09806	0.12269	0.80884	0.0095
		Expr_quant	-1.21336	0.2972	0.58311	0.0375
	TNXB	Pathologic_stage	0.56011	1.75086	0.2683	0.0368
OS	DIRAS1	Pathologic_stage	1.12604	3.08344	0.29716	0.0002
		Radiation_therapy	-3.9416	0.01942	1.08224	0.0003
	IL22RA2	Pathologic_stage	0.9143	2.49502	0.42703	0.0323
	MICA	Expression	4.936,011	139.214	1.840,017	0.0073
		Pathologic_stage	1.125,939	3.08311	0.275,097	4E-05
		Radiation_therapy	-3.643,406	0.02616	1.172,328	0.0019
		Expr_quant	-1.656,961	0.19072	0.803,941	0.0393
	S100B	Pathologic_stage	0.873,639	2.39561	0.23499	0.0002
		Radiation_therapy	-3.068291	0.0465	0.944,318	0.0012

### Analysis of the 94 DEGs in other studies highlight similar DEGs as possible biomarkers

Finally, to evaluate whether the data from GSE162187 (94 DEGs between resistant and sensitive patients) has a consistent expression with other studies, we analyzed the data of the study GSE163882, which aimed to predict pCR to neoadjuvant therapy in breast cancer patients. Data from GSE162187 were used as training data, and data from the GSE163882 dataset were used as corroboration data. This analysis discard 84 DEGs and identified 10 DEGs in common (ATF6B, ERAL1, CRYM, MUC16, SOX10, MICA, PDE2A, TMEM97, SDF2, and BICDL2) that could discriminate patient outcomes. Therefore, these 10 DEGs were considered possible biomarkers of pCR and neoadjuvant chemotherapy response. Moreover, three DEGs (ATF6B, ERAL1, and MICA) have a strong correlation with DFS and OS.

## Discussion

One fundamental aspect of treating breast cancer patients is the knowledge of their molecular subtypes. This information has *per se* a prognostic value for predicting patient treatment response (von Minckwitz et al., 2012), which can be evaluated according to the criteria for the diagnosis of pCR. Achieving pCR has been associated with better overall survival (Broglio et al., 2016; Spring et al., 2020); however, the intrinsic factors involved in pCR have not been clarified. There is still controversy on whether standard adjuvant therapy increases pCR (Mauri et al., 2005). The percentage of patients who achieve pCR ranges between 27%–47% (Muller et al., 2021; Xin et al., 2021). In this study, 37.5% of patients achieved pCR. Therefore, HER2+ breast cancer patients were categorized as sensitive (pCR achieved) or resistant (pCR did not achieve) to neoadjuvant chemotherapy and were used as an RNA-seq strategy to identify predictors of pCR. It should be considered that the diagnosis of HER2+ breast cancer was because more than 10% of the tumor cells present detectable HER2 expression; therefore, there is a large percentage of cells that do not express HER2. This highlights the heterogeneity of this breast cancer subtype (Ng et al., 2015; Chen et al., 2020). When the transcriptional profiles of all patients were compared to determine clusters, this heterogeneity was emphasized (Supplementary Figure S1A). The implications of molecular differences in HER2+ breast cancer patients are not fully understood and may be relevant to prognosis and treatment response.

The DEGs found in HER2+ breast cancer patients sensitive and resistant to neoadjuvant chemotherapy were mainly related to plasma membranes, vesicles, and extracellular space and were involved in different biological processes, such as cellular response to chemical stimulus, cell adhesion, and signal regulation. Variations in the protein components of the extracellular matrix have been reported in breast tumors of different origins (Borghesi et al., 2021). In addition, extracellular components such as the extracellular matrix, vesicles, and plasma membranes can be modified by cancer-associated fibroblasts, leading to a tumor microenvironment involved in cancer development and drug resistance (Mashouri et al., 2019; Helal-Neto et al., 2020; Lugo-Cintron et al., 2020).

One of the most enriched pathways is related to cell adhesion molecules involved in tight junctions of epithelial and endothelial cells, such as claudins, which participate in epithelial-mesenchymal transition (EMT) and chemoresistance (Hewitt et al., 2006; Agarwal et al., 2009; Gowrikumar et al., 2019). According to KEGG enrichment analysis, the AMPK signaling pathway is involved in the resistance process; this pathway is considered a double-edged sword that protects and promotes cancer progression (Jeon and Hay, 2015). Sensitization of breast cancer cells to chemotherapy by activating AMPK signaling by CTAB has been observed (Pan et al., 2019). Similarly, histological evaluations have reported altered AMPK signaling in breast cancer samples (Hadad et al., 2009), and this pathway is considered a therapeutic target for breast cancer treatment (Hadad et al., 2008). However, it has been hypothesized that once cancer has developed, AMPK promotes the survival of cancer cells by protecting them against DNA damage, nutritional stress, and hypoxia (Russell and Hardie, 2020). Further studies are needed to delineate the role of the AMPK pathway in breast cancer and the development of chemotherapy resistance.

Another enriched pathway was cyclic guanosine 3,5-monophosphate (cGMP) and protein kinase G (PKG). The cGMP-PKG pathway has been associated with the modulation of apoptosis and growth inhibition in MCF-7 and MDA-MB-468 breast cancer cell lines (Fallahian et al., 2011). An essential component of this pathway is the protein kinase cGMP-dependent 2 (PRKG2), which was found to be downregulated in the resistant group in this study. Our results correlate with those of Karami-Tehrani et al. (Karami-Tehrani et al., 2012), who observed lower expression of PRKG2 protein in breast tumor samples. In addition, it has been reported that PRKG2 inhibits EGF-induced MAPK/c-Jun N-terminal kinase (JNK) signal transduction in human breast cancer cells (Lan et al., 2012) and also inhibits the activation of EGFR and HER2 in gastric cancer cells (Zhu et al., 2016; Lan et al., 2019). PRKG2 inhibits the migration, invasion, and proliferation of cancer cells and activates CREB, which modulates anti-apoptotic genes, such as BCL2 (Shankar et al., 2010), which are overexpressed in the resistant group, thereby contributing to the survival of cancer cells in the resistant group.

In this study, many DEGs related to resistance were identified. With the dimensional reduction, samples clustered better, highlighting the possibility of using these genes to predict the response to treatment. An interesting finding in our results was a group of DEGs that interacted with each other, including HLA-A, HLA-DQA1, HLA-DRB1, HLA-B, and TRIM26, which are components of the MHC protein complex, except TRIM26. These DEGs were found to be overexpressed in the resistant group. The upregulation of classical and non-classical HLA-I molecules has been reported to acquire a “protective” phenotype in melanoma cells (Balsamo et al., 2012). HLA molecules play a role in self-recognition by immune cells, which is essential for hematopoietic and healthy cells to avoid their destruction, and the loss, alteration, or absence of HLA molecules can cause susceptibility to NK cell attack (Ljunggren and Karre, 1990; Moretta et al., 2004). HLA molecules interact with inhibitory receptors such as killer cell immunoglobulin-like receptors (KIRs), leukocyte immunoglobulin-like receptors (LIRs), and natural killer group 2A (NKG2A) on the NK surface, avoiding its activation (Khan et al., 2020). Overexpression of HLA by cancer cells has been reported as a mechanism for evading the immune response of NK cells and is termed immune checkpoint inhibition (Bi and Tian, 2019). From this group of genes, a variant of HLA-A (ENSEMBL ID ENSG00000235657) was observed subexpressed in the resistance group. In addition, low expression of this gene was associated with a worse prognosis for OS. This gene has already been reported to predict treatment response and OS (Sinn et al., 2021; Barron-Gallardo et al., 2022).

ATF6B has two ensemble IDs (ENSG00000228628 and ENSG00000213676). ENSG00000228628 ID was overexpressed. Nevertheless, ENSG00000213676, which corresponds to the primary assembly of this gene, was found to be sub-expressed, and low expression was related to lower OS and worse DFS. Variants of this gene have been associated with an increased risk of breast cancer development (Dierssen-Sotos et al., 2018).

DIRAS1 was found to be sub-expressed in the resistance group. Subexpression of this gene was correlated with lower OS. This gene has tumor-suppressive activity by binding to SmgGDS, which blocks the interactions of small GTPases, such as Rho and K-Ras4B. The expression of DIRAS1 is downregulated in most types of breast cancer (Bergom et al., 2016).

Other sub-expressed genes in resistant treatment and lower DFS were GRIN2B, GNG4, and IRX3. GRIN2B is involved in breast cancer progression and acts as a promoter of CpG islands (Park et al., 2011; Park et al., 2012). GNG4 is hypermethylated in breast cancer; however, when comparing all molecular subtypes, the HER2 subtype shows the highest expression levels for this gene (Fernandez-Nogueira et al., 2016; Mao et al., 2021). IRX3 plays an important role in obesity and type 2 diabetes; however, it plays an important role in the adaptability of tumor cells to metabolic challenges, a process that has a parallelism with the development of chemotherapeutic resistance (Singh et al., 2016).

A set of genes that showed high expression in the resistant group, which were related to lower OS and worse DFS, were L1CAM, MICA, and TNXB. The expression of L1CAM is increased in luminal B breast cancer, and its expression is related to disease recurrence and higher levels of Ki-67 expression (Moisini et al., 2021). A soluble form of L1CAM has been found in HER2-enriched primary breast cancer patients (Wu et al., 2018). There are reports that inhibition of L1CAM reverses cisplatin resistance in triple-negative breast cancer cells (Zhang et al., 2022). MICA is overexpressed in breast cancer when compared to normal tissue and is considered an indicator of poor prognosis (Madjd et al., 2007). It is an activation ligand of NK cells, which induces the lysis of cells that express it. However, there is a soluble form of MICA (sMICA) that decreases the expression and presentation of NKG2D, a natural cytotoxic receptor in natural killer cells, thus sMICA helps cancer cells to evade immune cell attack (Pan et al., 2017) and contributing to a worse prognosis in cancer (Roshani et al., 2016). In this study, high expression of MICA was observed in the resistant and lower OS groups; however, further studies are needed to determine the role of MICA or sMICA in chemotherapy resistance. In the case of TNXB, the expression of this gene has been analyzed in breast cancer, and a correlation between high TNXB expression and good survival prognosis has been found (Liot et al., 2020). Its expression decreases at late stages, major tumor grade, and node status of the disease (Liot et al., 2020), however, its expression in the HER2 molecular subtype and in relation to chemotherapy resistance has not been evaluated.

In contrast, genes with high expression but related to better OS and DFS were IL22RA2, PRTFDC1, PBX2, S100B, SERPINF2, DHRS13, ERAL1, and SLC9A3R2. IL22RA2 expression decreases in luminal A, B, and triple-negative breast cancers (Fu et al., 2015); however, but HER2+ breast cancer has not been reported. PRTFDC1 has been associated with the triple-negative basal-like immune-suppressed breast cancer subtype (TNBC-BLIS), which is considered one of the worst prognoses (Yin et al., 2020). The most highly expressed gene is PBX2. This gene was found to be upregulated in breast lesions and has been proposed along with other genes as a candidate biomarker for distinguishing breast cancer lesions (Hou et al., 2020). It has been showed that the overexpression of PBX2 increases the tumorigenic properties of SkBr3 breast cancer cell line when transfected with HoxB7 (Fernandez et al., 2008). S100B expression has been negatively correlated with lymph node metastasis (Wang et al., 2021), inhibition of cell migration, better overall survival in luminal B breast cancer patients, and being a good distant metastases-free survival biomarker (Yen et al., 2018). SERPINF2 is differentially expressed in breast cancer tissues compared with normal tissues (Malvia et al., 2019). The protein product of SERPINF2 has been found in the serum of breast cancer patients when evaluating treatment response; however, this protein appeared in both resistant and sensitive groups (Chantada-Vazquez et al., 2022).

Finally, DHRS13, ERAL1, and SLC9A3R2 could predict treatment response and survival; however, there are no reports related to breast cancer and its possible function in this disease.

## Conclusion

This study underlines a molecular expression pattern related to the response of patients with HER2-positive breast cancer to neoadjuvant chemotherapy. Differentially expressed genes highlight the involvement of pathways, such as extracellular components, adhesion molecules, and immune responses, in the process of resistance to chemotherapy. Some differentially expressed genes can be used as biomarkers of overall survival and disease-free survival in breast cancers.

## Data Availability

The datasets presented in this study can be found in online repositories. The names of the repository/repositories and accession number(s) can be found in the article/Supplementary Material.

## References

[B1] Abal M. Andreu J. M. Barasoain I. (2003). Taxanes: Microtubule and centrosome targets, and cell cycle dependent mechanisms of action. Curr. Cancer Drug Targets 3 (3), 193–203. 10.2174/1568009033481967 12769688

[B2] Agarwal R. Mori Y. Cheng Y. Jin Z. Olaru A. V. Hamilton J. P. (2009). Silencing of claudin-11 is associated with increased invasiveness of gastric cancer cells. PLoS One 4 (11), e8002. 10.1371/journal.pone.0008002 19956721 PMC2776495

[B3] Balsamo M. Vermi W. Parodi M. Pietra G. Manzini C. Queirolo P. (2012). Melanoma cells become resistant to NK-cell-mediated killing when exposed to NK-cell numbers compatible with NK-cell infiltration in the tumor. Eur. J. Immunol. 42 (7), 1833–1842. 10.1002/eji.201142179 22585684

[B4] Ban M. Petric Mise B. Vrdoljak E. (2020). Early HER2-positive breast cancer: Current treatment and novel approaches. Breast Care (Basel) 15 (6), 560–569. 10.1159/000511883 33447229 PMC7768133

[B5] Barron-Gallardo C. A. Garcia-Chagollan M. Moran-Mendoza A. J. Delgadillo-Cristerna R. Martinez-Silva M. G. Aguilar-Lemarroy A. (2022). Transcriptomic analysis of breast cancer patients sensitive and resistant to chemotherapy: Looking for overall survival and drug resistance biomarkers. Technol. Cancer Res. Treat. 21, 15330338211068965. 10.1177/15330338211068965 34981997 PMC8733364

[B6] Bergom C. Hauser A. D. Rymaszewski A. Gonyo P. Prokop J. W. Jennings B. C. (2016). The tumor-suppressive small GTPase DiRas1 binds the noncanonical guanine nucleotide exchange factor SmgGDS and antagonizes SmgGDS interactions with oncogenic small GTPases. J. Biol. Chem. 291 (12), 6534–6545. 10.1074/jbc.M115.696831 26814130 PMC4813585

[B7] Bi J. Tian Z. (2019). NK cell dysfunction and checkpoint immunotherapy. Front. Immunol. 10, 1999. 10.3389/fimmu.2019.01999 31552017 PMC6736636

[B8] Borghesi J. Giancoli Kato Cano da Silva M. de Oliveira Pimenta Guimaraes K. Mario L. C. de Almeida da Anunciacao A. R. Silveira Rabelo A. C. (2021). Evaluation of immunohistopathological profile of tubular and solid canine mammary carcinomas. Res. Vet. Sci. 136, 119–126. 10.1016/j.rvsc.2021.02.004 33609969

[B9] Bray N. L. Pimentel H. Melsted P. Pachter L. (2016). Near-optimal probabilistic RNA-seq quantification. Nat. Biotechnol. 34 (5), 525–527. 10.1038/nbt.3519 27043002

[B10] Broglio K. R. Quintana M. Foster M. Olinger M. McGlothlin A. Berry S. M. (2016). Association of pathologic complete response to neoadjuvant therapy in HER2-positive breast cancer with long-term outcomes: A meta-analysis. JAMA Oncol. 2 (6), 751–760. 10.1001/jamaoncol.2015.6113 26914222

[B11] Chantada-Vazquez M. D. P. Conde-Amboage M. Grana-Lopez L. Vazquez-Estevez S. Bravo S. B. Nunez C. (2022). Circulating proteins associated with response and resistance to neoadjuvant chemotherapy in HER2-positive breast cancer. Cancers (Basel) 14 (4), 1087. 10.3390/cancers14041087 35205837 PMC8870308

[B12] Chen B. Zhang G. Wei G. Wang Y. Guo L. Lin J. (2020). Heterogeneity of genomic profile in patients with HER2-positive breast cancer. Endocr. Relat. Cancer 27 (3), 153–162. 10.1530/ERC-19-0414 31905165

[B13] Dierssen-Sotos T. Palazuelos-Calderon C. Jimenez-Moleon J. J. Aragones N. Altzibar J. M. Castano-Vinyals G. (2018). Reproductive risk factors in breast cancer and genetic hormonal pathways: A gene-environment interaction in the MCC-Spain project. BMC Cancer 18 (1), 280. 10.1186/s12885-018-4182-3 29530003 PMC5848450

[B14] Dodt M. Roehr J. T. Ahmed R. Dieterich C. (2012). FLEXBAR-flexible barcode and adapter processing for next-generation sequencing platforms. Biol. (Basel) 1 (3), 895–905. 10.3390/biology1030895 PMC4009805 24832523

[B15] Fallahian F. Karami-Tehrani F. Salami S. Aghaei M. (2011). Cyclic GMP induced apoptosis via protein kinase G in oestrogen receptor-positive and -negative breast cancer cell lines. FEBS J. 278 (18), 3360–3369. 10.1111/j.1742-4658.2011.08260.x 21777390

[B16] Fernandez L. C. Errico M. C. Bottero L. Penkov D. Resnati M. Blasi F. (2008). Oncogenic HoxB7 requires TALE cofactors and is inactivated by a dominant-negative Pbx1 mutant in a cell-specific manner. Cancer Lett. 266 (2), 144–155. 10.1016/j.canlet.2008.02.042 18378073

[B17] Fernandez-Nogueira P. Bragado P. Almendro V. Ametller E. Rios J. Choudhury S. (2016). Differential expression of neurogenes among breast cancer subtypes identifies high risk patients. Oncotarget 7 (5), 5313–5326. 10.18632/oncotarget.6543 26673618 PMC4868688

[B18] Fu J. Khaybullin R. Zhang Y. Xia A. Qi X. (2015). Gene expression profiling leads to discovery of correlation of matrix metalloproteinase 11 and heparanase 2 in breast cancer progression. BMC Cancer 15, 473. 10.1186/s12885-015-1410-y 26084486 PMC4477316

[B19] Gowrikumar S. Singh A. B. Dhawan P. (2019). Role of claudin proteins in regulating cancer stem cells and chemoresistance-potential implication in disease prognosis and therapy. Int. J. Mol. Sci. 21 (1), E53. 10.3390/ijms21010053 31861759 PMC6982342

[B20] Hadad S. M. Baker L. Quinlan P. R. Robertson K. E. Bray S. E. Thomson G. (2009). Histological evaluation of AMPK signalling in primary breast cancer. BMC Cancer 9, 307. 10.1186/1471-2407-9-307 19723334 PMC2744705

[B21] Hadad S. M. Fleming S. Thompson A. M. (2008). Targeting AMPK: A new therapeutic opportunity in breast cancer. Crit. Rev. Oncol. Hematol. 67 (1), 1–7. 10.1016/j.critrevonc.2008.01.007 18343152

[B22] Harbeck N. Gnant M. (2017). Breast cancer. Lancet 389 (10074), 1134–1150. 10.1016/S0140-6736(16)31891-8 27865536

[B23] Helal-Neto E. Barcellos-de-Souza P. Morgado-Diaz J. Barja-Fidalgo C. Barja-Fidalgo C. (2020). Extracellular matrix derived from high metastatic human breast cancer triggers epithelial-mesenchymal transition in epithelial breast cancer cells through αvβ3 integrin. Int. J. Mol. Sci. 21 (8), E2995. 10.3390/ijms21082995 32340328 PMC7216035

[B24] Hewitt K. J. Agarwal R. Morin P. J. (2006). The claudin gene family: Expression in normal and neoplastic tissues. BMC Cancer 6, 186. 10.1186/1471-2407-6-186 16836752 PMC1538620

[B25] Hou H. Lyu Y. Jiang J. Wang M. Zhang R. Liew C. C. (2020). Peripheral blood transcriptome identifies high-risk benign and malignant breast lesions. PLoS One 15 (6), e0233713. 10.1371/journal.pone.0233713 32497068 PMC7272048

[B26] Iwamoto T. Kajiwara Y. Zhu Y. Iha S. (2020). Biomarkers of neoadjuvant/adjuvant chemotherapy for breast cancer. Chin. Clin. Oncol. 9 (3), 27. 10.21037/cco.2020.01.06 32192349

[B27] Jeon S. M. Hay N. (2015). The double-edged sword of AMPK signaling in cancer and its therapeutic implications. Arch. Pharm. Res. 38 (3), 346–357. 10.1007/s12272-015-0549-z 25575627 PMC5789788

[B28] Kanehisa M. Sato Y. (2020). KEGG Mapper for inferring cellular functions from protein sequences. Protein Sci. 29 (1), 28–35. 10.1002/pro.3711 31423653 PMC6933857

[B29] Karami-Tehrani F. Fallahian F. Atri M. (2012). Expression of cGMP-dependent protein kinase, PKGIα, PKGIβ, and PKGII in malignant and benign breast tumors. Tumour Biol. 33 (6), 1927–1932. 10.1007/s13277-012-0453-9 22791569

[B30] Khan M. Arooj S. Wang H. (2020). NK cell-based immune checkpoint inhibition. Front. Immunol. 11, 167. 10.3389/fimmu.2020.00167 32117298 PMC7031489

[B31] Lan T. Chen Y. Sang J. Wu Y. Wang Y. Jiang L. (2012). Type II cGMP-dependent protein kinase inhibits EGF-induced MAPK/JNK signal transduction in breast cancer cells. Oncol. Rep. 27 (6), 2039–2044. 10.3892/or.2012.1726 22427012

[B32] Lan T. Pang J. Wang Z. Wang Y. Qian H. Chen Y. (2019). Type II cGMP-dependent protein kinase phosphorylates EGFR at threonine 669 and thereby inhibits its activation. Biochem. Biophys. Res. Commun. 518 (1), 14–18. 10.1016/j.bbrc.2019.07.126 31395339

[B33] Liberzon A. Birger C. Thorvaldsdottir H. Ghandi M. Mesirov J. P. Tamayo P. (2015). The Molecular Signatures Database (MSigDB) hallmark gene set collection. Cell Syst. 1 (6), 417–425. 10.1016/j.cels.2015.12.004 26771021 PMC4707969

[B34] Liberzon A. Subramanian A. Pinchback R. Thorvaldsdottir H. Tamayo P. Mesirov J. P. (2011). Molecular signatures database (MSigDB) 3.0. Bioinformatics 27 (12), 1739–1740. 10.1093/bioinformatics/btr260 21546393 PMC3106198

[B35] Liot S. Aubert A. Hervieu V. Kholti N. E. Schalkwijk J. Verrier B. (2020). Loss of tenascin-X expression during tumor progression: A new pan-cancer marker. Matrix Biol. Plus 6-7, 100021. 10.1016/j.mbplus.2020.100021 33543019 PMC7852205

[B36] Ljunggren H. G. Karre K. (1990). In search of the 'missing self': MHC molecules and NK cell recognition. Immunol. Today 11 (7), 237–244. 10.1016/0167-5699(90)90097-s 2201309

[B37] Love M. I. Huber W. Anders S. (2014). Moderated estimation of fold change and dispersion for RNA-seq data with DESeq2. Genome Biol. 15 (12), 550. 10.1186/s13059-014-0550-8 25516281 PMC4302049

[B38] Lugo-Cintron K. M. Gong M. M. Ayuso J. M. Tomko L. A. Beebe D. J. Virumbrales-Munoz M. (2020). Breast fibroblasts and ECM components modulate breast cancer cell migration through the secretion of MMPs in a 3D microfluidic Co-culture model. Cancers (Basel) 12 (5), E1173. 10.3390/cancers12051173 32384738 PMC7281408

[B39] Madjd Z. Spendlove I. Moss R. Bevin S. Pinder S. E. Watson N. F. (2007). Upregulation of MICA on high-grade invasive operable breast carcinoma. Cancer Immun. 7, 17. 17948965 PMC2935745

[B40] Malvia S. Bagadi S. A. R. Pradhan D. Chintamani C. Bhatnagar A. Arora D. (2019). Study of gene expression profiles of breast cancers in Indian women. Sci. Rep. 9 (1), 10018. 10.1038/s41598-019-46261-1 31292488 PMC6620270

[B41] Mao X. H. Ye Q. Zhang G. B. Jiang J. Y. Zhao H. Y. Shao Y. F. (2021). Identification of differentially methylated genes as diagnostic and prognostic biomarkers of breast cancer. World J. Surg. Oncol. 19 (1), 29. 10.1186/s12957-021-02124-6 33499882 PMC7839189

[B42] Mashouri L. Yousefi H. Aref A. R. Ahadi A. M. Molaei F. Alahari S. K. (2019). Exosomes: Composition, biogenesis, and mechanisms in cancer metastasis and drug resistance. Mol. Cancer 18 (1), 75. 10.1186/s12943-019-0991-5 30940145 PMC6444571

[B43] Mauri D. Pavlidis N. Ioannidis J. P. (2005). Neoadjuvant versus adjuvant systemic treatment in breast cancer: A meta-analysis. J. Natl. Cancer Inst. 97 (3), 188–194. 10.1093/jnci/dji021 15687361

[B44] Maximiano S. Magalhaes P. Guerreiro M. P. Morgado M. (2016). Trastuzumab in the treatment of breast cancer. BioDrugs 30 (2), 75–86. 10.1007/s40259-016-0162-9 26892619

[B45] McGowan J. V. Chung R. Maulik A. Piotrowska I. Walker J. M. Yellon D. M. (2017). Anthracycline chemotherapy and cardiotoxicity. Cardiovasc. Drugs Ther. 31 (1), 63–75. 10.1007/s10557-016-6711-0 28185035 PMC5346598

[B46] Mi H. Ebert D. Muruganujan A. Mills C. Albou L. P. Mushayamaha T. (2021). PANTHER version 16: A revised family classification, tree-based classification tool, enhancer regions and extensive API. Nucleic Acids Res. 49 (D1), D394–D403. 10.1093/nar/gkaa1106 33290554 PMC7778891

[B47] Moisini I. Zhang H. D'Aguiar M. Hicks D. G. Turner B. M. (2021). L1CAM expression in recurrent estrogen positive/HER2 negative breast cancer: A novel biomarker worth considering. Appl. Immunohistochem. Mol. Morphol. 29 (4), 287–292. 10.1097/PAI.0000000000000909 33818537

[B48] Moretta L. Bottino C. Pende D. Vitale M. Mingari M. C. Moretta A. (2004). Different checkpoints in human NK-cell activation. Trends Immunol. 25 (12), 670–676. 10.1016/j.it.2004.09.008 15530838

[B49] Muller C. Schmidt G. Juhasz-Boss I. Jung L. Huwer S. Solomayer E. F. (2021). Influences on pathologic complete response in breast cancer patients after neoadjuvant chemotherapy. Arch. Gynecol. Obstet. 304 (4), 1065–1071. 10.1007/s00404-021-06018-6 33689016 PMC8429372

[B50] Ng C. K. Martelotto L. G. Gauthier A. Wen H. C. Piscuoglio S. Lim R. S. (2015). Intra-tumor genetic heterogeneity and alternative driver genetic alterations in breast cancers with heterogeneous HER2 gene amplification. Genome Biol. 16, 107. 10.1186/s13059-015-0657-6 25994018 PMC4440518

[B51] Pan J. Shen J. Si W. Du C. Chen D. Xu L. (2017). Resveratrol promotes MICA/B expression and natural killer cell lysis of breast cancer cells by suppressing c-Myc/miR-17 pathway. Oncotarget 8 (39), 65743–65758. 10.18632/oncotarget.19445 29029468 PMC5630368

[B52] Pan Y. Zhang Y. Chen Q. Tao X. Liu J. Xiao G. G. (2019). CTAB enhances chemo-sensitivity through activation of AMPK signaling cascades in breast cancer. Front. Pharmacol. 10, 843. 10.3389/fphar.2019.00843 31402869 PMC6676472

[B53] Park S. Y. Kwon H. J. Choi Y. Lee H. E. Kim S. W. Kim J. H. (2012). Distinct patterns of promoter CpG island methylation of breast cancer subtypes are associated with stem cell phenotypes. Mod. Pathol. 25 (2), 185–196. 10.1038/modpathol.2011.160 22037257

[B54] Park S. Y. Kwon H. J. Lee H. E. Ryu H. S. Kim S. W. Kim J. H. (2011). Promoter CpG island hypermethylation during breast cancer progression. Virchows Arch. 458 (1), 73–84. 10.1007/s00428-010-1013-6 21120523

[B55] Perou C. M. Sorlie T. Eisen M. B. van de Rijn M. Jeffrey S. S. Rees C. A. (2000). Molecular portraits of human breast tumours. Nature 406 (6797), 747–752. 10.1038/35021093 10963602

[B56] Roehr J. T. Dieterich C. Reinert K. (2017). Flexbar 3.0 - SIMD and multicore parallelization. Bioinformatics 33 (18), 2941–2942. 10.1093/bioinformatics/btx330 28541403

[B57] Roshani R. Boroujerdnia M. G. Talaiezadeh A. H. Khodadadi A. (2016). Assessment of changes in expression and presentation of NKG2D under influence of MICA serum factor in different stages of breast cancer. Tumour Biol. 37 (5), 6953–6962. 10.1007/s13277-015-4584-7 26662806

[B58] Russell F. M. Hardie D. G. (2020). AMP-activated protein kinase: Do we need activators or inhibitors to treat or prevent cancer? Int. J. Mol. Sci. 22 (1), E186. 10.3390/ijms22010186 33375416 PMC7795930

[B59] Shankar E. Krishnamurthy S. Paranandi R. Basu A. (2010). PKCepsilon induces Bcl-2 by activating CREB. Int. J. Oncol. 36 (4), 883–888. 10.3892/ijo_00000566 20198332

[B60] Singh B. Kinne H. E. Milligan R. D. Washburn L. J. Olsen M. Lucci A. (2016). Important role of FTO in the survival of rare panresistant triple-negative inflammatory breast cancer cells facing a severe metabolic challenge. PLoS One 11 (7), e0159072. 10.1371/journal.pone.0159072 27390851 PMC4938613

[B61] Sinn B. V. Loibl S. Hanusch C. A. Zahm D. M. Sinn H. P. Untch M. (2021). Immune-related gene expression predicts response to neoadjuvant chemotherapy but not additional benefit from PD-L1 inhibition in women with early triple-negative breast cancer. Clin. Cancer Res. 27 (9), 2584–2591. 10.1158/1078-0432.CCR-20-3113 33593886

[B62] Sorlie T. Tibshirani R. Parker J. Hastie T. Marron J. S. Nobel A. (2003). Repeated observation of breast tumor subtypes in independent gene expression data sets. Proc. Natl. Acad. Sci. U. S. A. 100 (14), 8418–8423. 10.1073/pnas.0932692100 12829800 PMC166244

[B63] Spring L. M. Fell G. Arfe A. Sharma C. Greenup R. Reynolds K. L. (2020). Pathologic complete response after neoadjuvant chemotherapy and impact on breast cancer recurrence and survival: A comprehensive meta-analysis. Clin. Cancer Res. 26 (12), 2838–2848. 10.1158/1078-0432.CCR-19-3492 32046998 PMC7299787

[B64] Subramanian A. Tamayo P. Mootha V. K. Mukherjee S. Ebert B. L. Gillette M. A. (2005). Gene set enrichment analysis: A knowledge-based approach for interpreting genome-wide expression profiles. Proc. Natl. Acad. Sci. U. S. A. 102 (43), 15545–15550. 10.1073/pnas.0506580102 16199517 PMC1239896

[B65] Sung H. Ferlay J. Siegel R. L. Laversanne M. Soerjomataram I. Jemal A. (2021). Global cancer statistics 2020: GLOBOCAN estimates of incidence and mortality worldwide for 36 cancers in 185 countries. Ca. Cancer J. Clin. 1, 209–249. 10.3322/caac.21660 33538338

[B66] Szklarczyk D. Gable A. L. Lyon D. Junge A. Wyder S. Huerta-Cepas J. (2019). STRING v11: Protein-protein association networks with increased coverage, supporting functional discovery in genome-wide experimental datasets. Nucleic Acids Res. 47 (D1), D607–D613. 10.1093/nar/gky1131 30476243 PMC6323986

[B67] von Minckwitz G. Untch M. Blohmer J. U. Costa S. D. Eidtmann H. Fasching P. A. (2012). Definition and impact of pathologic complete response on prognosis after neoadjuvant chemotherapy in various intrinsic breast cancer subtypes. J. Clin. Oncol. 30 (15), 1796–1804. 10.1200/JCO.2011.38.8595 22508812

[B68] Waks A. G. Winer E. P. (2019). Breast cancer treatment: A review. JAMA 321 (3), 288–300. 10.1001/jama.2018.19323 30667505

[B69] Wang C. Xu K. Deng F. Liu Y. Huang J. Wang R. (2021). A six-gene signature related with tumor mutation burden for predicting lymph node metastasis in breast cancer. Transl. Cancer Res. 10 (5), 2229–2246. 10.21037/tcr-20-3471 35116541 PMC8798002

[B70] Wu J. D. Hong C. Q. Huang W. H. Wei X. L. Zhang F. Zhuang Y. X. (2018). L1 cell adhesion molecule and its soluble form sL1 exhibit poor prognosis in primary breast cancer patients. Clin. Breast Cancer 18 (5), e851–e861. 10.1016/j.clbc.2017.12.011 29510897

[B71] Xin L. Zhang H. Zhang S. Cheng Y. J. Liu Q. Xu L. (2021). [Docetaxel, carboplatin plus trastuzumab as neoadjuvant setting in patients with early-stage human epidermal growth factor receptor 2 positive breast cancer: A retrospective analysis]. Zhonghua Wai Ke Za Zhi 59 (3), 222–227. 10.3760/cma.j.cn112139-20201122-00811 33685057

[B72] Yardley D. A. (2013). nab-Paclitaxel mechanisms of action and delivery. J. Control. Release 170 (3), 365–372. 10.1016/j.jconrel.2013.05.041 23770008

[B73] Yen M. C. Huang Y. C. Kan J. Y. Kuo P. L. Hou M. F. Hsu Y. L. (2018). S100B expression in breast cancer as a predictive marker for cancer metastasis. Int. J. Oncol. 52 (2), 433–440. 10.3892/ijo.2017.4226 29345293

[B74] Yin L. Duan J. J. Bian X. W. Yu S. C. (2020). Triple-negative breast cancer molecular subtyping and treatment progress. Breast Cancer Res. 22 (1), 61. 10.1186/s13058-020-01296-5 32517735 PMC7285581

[B75] Zhang L. Y. Shen Z. X. Guo L. (2022). Inhibiting L1CAM reverses cisplatin resistance of triple negative breast cancer cells by blocking AKT signaling pathway. Cancer Invest. 40 (4), 313–324. 10.1080/07357907.2021.2016801 35040385

[B76] Zhu M. Yao X. Wu M. Qian H. Wu Y. Chen Y. (2016). Type II cGMP-dependent protein kinase directly inhibits HER2 activation of gastric cancer cells. Mol. Med. Rep. 13 (2), 1909–1913. 10.3892/mmr.2015.4688 26676300

