# Supplementary material for: A gene expression signature in HER2+ breast cancer patients related to neoadjuvant chemotherapy resistance, overall survival, and disease-free survival
Source: Front Genet. 2022 Oct 21;13:991706. doi: 10.3389/fgene.2022.991706 (PMC9634254; doi:10.3389/fgene.2022.991706)
Supplement: Supplementary file 1 [file DataSheet2.PDF]

Supplementary Table 1. Clinical Characteristics of Patients

ID	Treatment Response	Age	Molecular Subtype	Histological Type	SBR
3	Resistant	49	HER2+	Invasive Ductal Carcinoma	SBRII
12	Resistant	65	HER2+	Invasive Ductal Carcinoma	SBRII
27	Resistant	56	HER2+	Invasive Ductal Carcinoma	NA
28	Resistant	33	HER2+	Invasive Ductal Carcinoma	SBRIII
44	Resistant	58	HER2+	Invasive Ductal Carcinoma	SBRII
7	Sensitive	64	HER2+	Invasive Ductal Carcinoma	SBRII
24	Sensitive	67	HER2+	Invasive Ductal Carcinoma	SBRIII
32	Sensitive	55	HER2+	Invasive Ductal Carcinoma	SBRIII
