# Supplementary material for: A gene expression signature in HER2+ breast cancer patients related to neoadjuvant chemotherapy resistance, overall survival, and disease-free survival
Source: Front Genet. 2022 Oct 21;13:991706. doi: 10.3389/fgene.2022.991706 (PMC9634254; doi:10.3389/fgene.2022.991706)
Supplement: Supplementary file 2 [file DataSheet4.PDF]

Table supplementary 3. DFS and OS odds-ratio

	Time (years)	Gene	Odd ratio	p-value
DFS	5	ATF6B	4.6	0.037 *
	5	DHRS13	9.9	0.005 *
	5	DIRAS1	5.0	0.027 *
	5	ERAL1	4.8	0.032 *
	5	GRIN2B	2.6	0.152
	5	IRX3	NA	NA
	5	L1CAM	0.19	0.027 *
	5	PBX2	4.4	0.044 *
	5	PRTFDC1	2.8	0.134
	10	S100B	NA	NA
	10	SLC9A3R2	0.29	0.069
	10	TNXB	0.12	0.032 *
OS	10	DIRAS1	6.0	0.009 *
	5	GNG4	NA	NA
	10	HLA-A	5.0	0.117
	5	IL22RA2	5.0	0.117
	5	MICA	0.20	0.027 *
	5	S100B	4.7	0.032 *
	5	SERPINF	NA	NA
	10	TNXB	0.14	0.048 *
Odds-ratio values greater than 1 refer to the odds that a patient with a high-expression gene comes alive or disease-free versus a patient with a low-expression gene. Time (years) is the period of OS (Overall Survival) or DFS (Disease free survival). Genes with NA odds-ratio presented zero cases of death or recurrence in any of the 2 groups of expression.				
