# Supplementary material for: A gene expression signature in HER2+ breast cancer patients related to neoadjuvant chemotherapy resistance, overall survival, and disease-free survival
Source: Front Genet. 2022 Oct 21;13:991706. doi: 10.3389/fgene.2022.991706 (PMC9634254; doi:10.3389/fgene.2022.991706)
Supplement: Supplementary file 3 [file DataSheet3.PDF]

Supplementary Table 2. DEGs with P-adj value <0.05

ENSEMBL_ID	Symbol	Log2(fold-change)	P-adj
ENSG00000228284	HLA-DQA1	23.5333173357261	7.51563140477073E-09
ENSG00000230230	TRIM26	21.2902628726626	4.07616000605446E-06
ENSG00000282797	IGHJ6	20.6872786743856	1.00226309772998E-05
ENSG00000228892	AGPAT1	10.5749288077991	0.004518339431978
ENSG00000236873	AGPAT1	10.5749288077991	0.004518339431978
ENSG00000275148	IGHV1-69-2	10.103408969182	0.03847312636592
ENSG00000281990	IGHV1-69-2	10.103408969182	0.03847312636592
ENSG00000237344	PBX2	9.7958044819393	0.034389114443364
ENSG00000228080	HLA-DRB1	8.71930546612371	0.00823650398971
ENSG00000206427	PRRC2A	7.71886310256083	2.75219478549584E-14
ENSG00000231825	PRRC2A	7.71886310256083	2.75219478549584E-14
ENSG00000225164	PRRC2A	7.6007584683403	2.0682709959139E-14
ENSG00000275621	LRRC37A3	7.31128192845233	0.022707687843169
ENSG00000236236	TNXB	7.27605701162602	0.022596713612255
ENSG00000100146	SOX10	6.29040936798872	0.003421868150058
ENSG00000276345	AC004556.3	6.08892454604164	0.02166006529295
ENSG00000181143	MUC16	5.32200984003897	0.00477947145453
ENSG00000211655	IGLV1-36	5.24008742751701	0.034342697147339
ENSG00000273737	IGLV1-36	5.24008742751701	0.034342697147339
ENSG00000131668	BARX1	4.71501339185014	0.041926663551924
ENSG00000211664	IGLV2-18	4.53583422446179	0.004032306727249
ENSG00000228628	ATF6B	4.4358192006686	0.009031543397877
ENSG00000223532	HLA-B	4.41267856897208	0.011071234824631
ENSG00000163017	ACTG2	4.2983199114699	0.02166006529295
ENSG00000150471	ADGRL3	4.27421374405249	0.040600005360739
ENSG00000164485	IL22RA2	4.25378572805503	0.031315973714055
ENSG00000231225	MICA	3.85720204184226	0.021372168486045
ENSG00000233051	MICA	3.85720204184226	0.021372168486045
ENSG00000112936	C7	3.74944970507917	0.000119196755169
ENSG00000147576	ADHFE1	3.49205946377984	0.005658410407382
ENSG00000073282	TP63	3.43446267357473	0.009678935979039
ENSG00000198910	L1CAM	3.42691539148045	0.00047014543061
ENSG00000101311	FERMT1	3.24022808741756	0.029022095770039

ENSG00000278828	H3C10	3.10667133502303	0.009032304307376
ENSG00000154263	ABCA10	3.09985336352915	0.001652992827293
ENSG00000206384	COL6A6	3.04157629810185	0.022596713612255
ENSG00000160307	S100B	2.8194988815828	0.004313104300606
ENSG00000196747	H2AC13	2.74595803487472	0.043950509680087
ENSG00000256229	ZNF486	2.53357061885069	0.044317514929833
ENSG00000162706	CADM3	2.52881344038938	0.003683599540058
ENSG00000156738	MS4A1	2.31757490552177	0.049932090904054
ENSG00000167711	SERPINF2	2.31232878556714	0.005489700860663
ENSG00000198774	RASSF9	2.29163043020516	0.044622509455949
ENSG00000099256	PRTFDC1	1.77289441143998	0.019339831118728
ENSG00000203814	H2BC18	1.76314843397281	0.03415951403572
ENSG00000186642	PDE2A	1.59194429465368	0.049932090904054
ENSG00000170903	MSANTD4	1.56847435233842	0.011071234824631
ENSG00000169508	GPR183	1.55876891555837	0.00768277619805
ENSG00000166025	AMOTL1	1.3828856908057	0.038766929077208
ENSG00000102100	SLC35A2	-1.24736429209656	0.006376828058485
ENSG00000100359	SGSM3	-1.24845076246839	0.007672371955913
ENSG00000132591	ERAL1	-1.38161469654705	0.013944941850221
ENSG00000167969	ECI1	-1.39743842359911	0.000905189762628
ENSG00000109084	TMEM97	-1.40775591148826	0.022498748035784
ENSG00000177508	IRX3	-1.48559743741302	0.003421868150058
ENSG00000198242	RPL23A	-1.50987475953525	0.005658410407382
ENSG00000171302	CANT1	-1.52140573959861	0.044317514929833
ENSG00000188322	SBK1	-1.52858123700693	0.02166006529295
ENSG00000132581	SDF2	-1.60641242377889	0.000241583433513
ENSG00000076864	RAP1GAP	-1.63537076716838	0.004032306727249
ENSG00000076604	TRAF4	-1.74775071108873	0.03847312636592
ENSG00000065054	SLC9A3R2	-1.81792543815886	0.048442798483006
ENSG00000160606	TLCD1	-1.90067004347118	0.016635742120469
ENSG00000168209	DDIT4	-1.96722686859879	0.002244266712738
ENSG00000205710	C17orf107	-2.14304048793248	0.023796961507526
ENSG00000162069	BICDL2	-2.17083468481253	0.0226910598747
ENSG00000108582	CPD	-2.24085590766466	0.029022095770039
ENSG00000176490	DIRAS1	-2.24460527946058	0.014501606880228

ENSG00000167536	DHRS13	-2.40523867285597	1.02754023800871E-06
ENSG00000184828	ZBTB7C	-2.60769271338503	0.049932090904054
ENSG00000177614	PGBD5	-2.61411167364323	0.016635742120469
ENSG00000162981	LRATD1	-2.66528015864151	0.007790118264795
ENSG00000069011	PITX1	-2.686829965192	0.046883531714842
ENSG00000277936	MRPL45	-2.95922273139831	0.03847312636592
ENSG00000183569	SERHL2	-2.98753846912734	0.030443480249544
ENSG00000158164	TMSB15A	-3.18169714507081	0.03415951403572
ENSG00000167771	RCOR2	-3.22495502450273	0.003421868150058
ENSG00000277363	SRCIN1	-3.32897184240831	0.049932090904054
ENSG00000109956	B3GAT1	-3.44360797846341	0.021372168486045
ENSG00000275861	TBC1D3I	-3.50339977460172	0.012840579508024
ENSG00000273608	SRCIN1	-3.52959974465585	0.035526288611535
ENSG00000103316	CRYM	-3.94296578271146	0.03775840914082
ENSG00000162078	ZG16B	-4.15746816946978	3.23011403593579E-05
ENSG00000283056	ZG16B	-4.15746816946978	3.23011403593579E-05
ENSG00000096006	CRISP3	-4.45684553302611	0.02166006529295
ENSG00000189001	SBSN	-4.52995708104357	0.038080674624464
ENSG00000168243	GNG4	-4.54726594760697	0.034668384712124
ENSG00000282972	GNG4	-4.54726594760697	0.034668384712124
ENSG00000124939	SCGB2A1	-4.73207376737116	0.020853848419618
ENSG00000273079	GRIN2B	-5.7214856919916	0.006458194078255
ENSG00000260220	CCDC187	-6.00358231052383	0.049932090904054
ENSG00000134184	GSTM1	-6.91364990123083	1.69299520793033E-10
ENSG00000214819	CDRT15L2	-9.19924969294971	0.033105529591458
ENSG00000235657	HLA-A	-10.3925546172358	4.38918285056899E-05
