# Supplementary material for: A gene expression signature in HER2+ breast cancer patients related to neoadjuvant chemotherapy resistance, overall survival, and disease-free survival
Source: Front Genet. 2022 Oct 21;13:991706. doi: 10.3389/fgene.2022.991706 (PMC9634254; doi:10.3389/fgene.2022.991706)
Supplement: Supplementary file 4 [file DataSheet1.PDF]

Supplementary Materials:

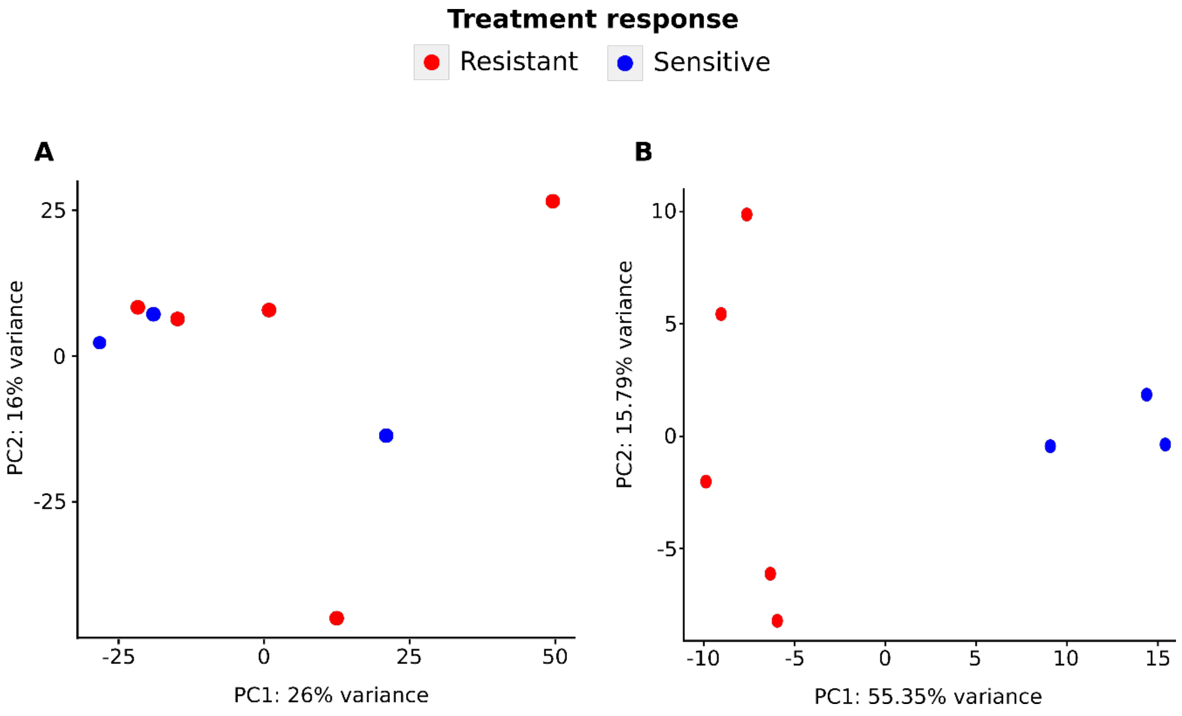

Supplementary Figure 1. Analysis of sample distribution by principal components according to gene expression. The PCA analysis was performed taking as input data **a)** the normalized read counts of all expressed genes, and **b)** the normalized read counts of DEGs with adjusted *P*-value <0.05. Each point represents a sample distributed according to principal components. The blue color indicates sensitive to treatment and the red color indicates resistance to treatment. The x-axis represents principal component 1 (PC1) and the y-axis represents principal component 2 (PC2).
